# Possible Gender Differences in the Level of Perceived Social Support in Couples Who Are Experiencing Issues With Infertility

**DOI:** 10.7759/cureus.29343

**Published:** 2022-09-19

**Authors:** Maya Pinzon, Shawna Rotoli

**Affiliations:** 1 Department of Molecular Biology, Rowan University School of Osteopathic Medicine, Stratford, USA

**Keywords:** experiences with infertility, psychological effects, gender differences, infertility, perceived social support

## Abstract

Objective

The purpose of this study is to examine whether there are gender differences in the level of perceived social support in couples experiencing issues with fertility.

Methods

A total of 938 participants aged 18-47 years, with self-reported issues of infertility, were evaluated using the Multidimensional Scale of Perceived Social Support (MSPSS) which comprises three subscales which correspond with distinct sources of social support: significant other (SO), family, and friends. Differences between sexes for total score and for all subscale scores were subsequently analyzed using SPSS Statistics (IBM Corp, Armonk, USA).

Results

Mean total scores and scores on all subscales (SO, family, friend) were higher in women (5.13 ± 1.10, 5.90 ± 1.20, 4.53 ± 1.53, 4.97 ± 1.40, respectively) compared with men (4.43 ± 1.58, 5.04 ± 1.90, 4.06 ± 1.76, 4.20 ± 1.81, respectively), a statistically significant difference of 0.70 (95% CI, -1.11 to -0.28), t(63.018) = -3.360, p = .001), 0.86 (95% CI, -1.35 to -0.36), t(62.277) = -3.452, p = .001, 0.47 (95% CI, -0.94 to -0.01), t(65.219) = -2.039, p = 0.046, 0.76 (95% CI, -1.24 to -0.29), t(63.018) = -3.360, p = 0.002), respectively. Males with male-factor infertility had a statistically significantly lower mean total score than males with female-factor infertility, -2.22 (95% CI, -3.71 to -0.74), p= 0.005. For males and females with male-factor infertility, mean “total” score was 2.73 (95% CI, 1.43 to 4.03) points lower for males than females, F(1,22) = 18.89, p < 0.001, partial η2 = 0.462.

Conclusion

Perceived social support among individuals experiencing issues with fertility was higher in females than in males across all subscales (SO, family, friends) with the biggest difference seen in SO score. Total scores differed with respect to infertility diagnosis in males but not in females, and amongst males and females with a male-factor infertility diagnosis, total scores were statistically significantly lower in males compared with females. Given the implications of high levels of perceived social support on improved overall well-being, our findings underscore the importance of implementing interventions that are focused on improving perceptions of social support in males experiencing issues with infertility, with specific consideration given to the unique experiences/ challenges and factors that may impact their experience.

## Introduction

Infertility is a global health issue [1] that often poses a physical, psychological, sociocultural, emotional, and financial burden on affected individuals [2]. Studies have shown that infertile couples experience significant anxiety and emotional distress, often describing it as “the most upsetting experience in their lives,” classifying it as either stressful or extremely stressful [3], with anxiety and depression being the most commonly reported mental health concerns [4]. Further, infertility is often seen as a silent struggle that is not openly discussed [5,6] due to associated feelings of shame, low self-esteem, fear that others won’t understand how they feel, and stigma due to social and cultural norms and values [7,8]. This secrecy in effect exacerbates/ evokes feelings of loneliness and isolation as individuals withdraw from sources of support, at a time when they paradoxically may need it the most [7]. In effect, this constellation of factors constitutes/creates significant challenges/obstacles for infertile individuals to maintain high levels of perceived social support.

The role of social support as a buffer in the context of numerous life stressors is well known [9]. It is thus of no surprise that social support has a positive effect on individuals experiencing issues with fertility [10], especially as a vulnerable population that is already at a higher risk of developing depression [5]. Specifically, studies suggest that higher perceptions of social support availability are associated with lower levels of general and infertility distress in both men and women [10,11]. 

Perceived social support is best conceptualized as a person’s subjective appraisal of his or her situation, rather than a true reflection of how much support is received [12].

In fact, perceived social support has been shown to be more predictive of health and more reliable in buffering against the adverse effects of stressors on psychological and physical well-being [13]. In a study of individuals with depression, greater perceived social support in contrast to greater received social support had a significantly larger relationship with lower depressive symptoms [12,14]. Moreover, perceived social support had a weak association with received support.

What is less understood is whether the perceptions of social support between the two sexes in the context of infertility differ. The literature on sex differences in the context of social support is mixed and inconsistent [13]. Given biomedical differences and differences in socialization processes and gender-role expectations [15], it is reasonable to anticipate differences in how infertility may be perceived and experienced between men and women. For example, male-factor infertility may be perceived as ‘inferior’ sperm quality and to some extent affect infertile males' perceptions of their masculinity [16]. For females, on the other hand, an inability to bear children and fulfill that ‘biological capability’ may challenge their core female identity and evoke intense fears of being blamed for the inability to give birth to a child [6,17]. Perhaps borne from this perception that motherhood is an intrinsic component of female nature and function, studies suggest that women were more likely to be labeled both by themselves and by others as responsible, regardless of which partner was actually infertile [17].

The purpose of this study is to examine whether there are differences in the level of perceived social support between sexes in the context of infertility. While some studies have investigated gender differences within couples with male-factor infertility and/or female-factor infertility [18], to our knowledge, there are no studies that focus on the personalization of the individual sexes’ perception of their social support. This study specifically aims to explore those gender differences in solely infertile individuals without regard to their partner (males with male-factor infertility vs. females with female-factor infertility. This understanding will provide insight and information for developing interventions to improve the experiences of infertile individuals.

## Materials and methods

Participants and study design

Participants were recruited through an anonymous Qualtrics survey (Qualtrics, North Sydney, Australia) posted to various online infertility support groups. All individuals above 18 years of age who reported experiencing issues with infertility (currently or prior) were eligible to participate in this observational, cross-sectional study. Participation in the study was voluntary and no individuals were excluded as long as they were at least 18 years of age. There was no cost nor compensation to the participants participating in this study. The study was approved by the Rowan University Institutional Review Board #Pro2020001151 (16 September 2021).

Survey tool

Participants who consented to this study completed a 24-question Qualtrics survey. Eight questions assessed demographics including: age, sex assigned at birth, race/ ethnicity, marital status, sexual orientation, gender identity, education level and household income. Fifteen questions assessed additional background information consisting of questions regarding relationships, pregnancies, infertility and social support network. The 12-item Multidimensional Scale of Perceived Social Support (MSPSS) [19] was used to assess participants’ level of perceived social support. The scale is comprised of three subscales which correspond with distinct sources of social support: significant other, family and friends (see Table 1).

**Table 1:** MSPSS Factors and Item Content MSPSS: Multidimensional Scale of Perceived Social Support

Family
My family really tries to help me
I get the emotional help and support I need from my family
I can talk about my problems with my family
My family is willing to help me make decisions

Friends
My friends really try to help me
I can count on my friends when things go wrong
I have friends with whom I can share my joys and sorrows
I can talk about my problems with my friends

Significant Other
There is a special person who is around when I am in need
There is a special person with whom I can share my joys and sorrows
I have a special person who is a real source of comfort to me
There is a special person in my life who cares about my feelings

Each subscale consists of four items with response options ranging from 1 (very strongly disagree) to 7 (very strongly agree). Higher scores indicate greater perceived social support. The MSPSS has been shown to be a valid measure of perceived social support and has demonstrated high internal consistency as well as reliability and validity with alpha values for the subscales and total scale between 85 and 91 [19]. Statistical analyses of survey results were conducted using IBM SPSS Statistics 28 (IBM Corp, Armonk, USA).

## Results

Demographic data were collected from participants and summarized in Table 2.

**Table 2:** Demographic Characteristics of Respondents (n= 938)

	Demographic Variables	n =938 (%)	Male sex at birth (n=60) (%)	Female sex at birth (n=878) (%)
Sex at birth	Male	60 (7.55)		
	Female	878 (92.45)		
Age range	18-23	15 (1.60)	2 (3.33)	13 (1.48)
	24-29	178 (18.98)	8 (13.33)	170 (19.36)
	30-35	508 (54.16)	26 (43.33)	482 (54.90)
	36-41	209 (22.28)	16 (26.67)	193 (21.98)
	42-47	28 (2.99)	8 (13.33)	20 (2.28)
Race/ ethnicity	White	801 (85.39)	42 (70.00)	759 (86.45)
	Hispanic or Latino	44 (4.69)	8 (13.33)	36 (4.10)
	Black or African American	16 (1.71)	3 (5.00)	13 (1.48)
	Native American or American Indian	2 (0.21)	0 (0)	2 (0.23)
	Asian or Pacific Islander	45 (4.80)	4 (6.67)	41 (4.67)
	Other	30 (3.20)	3 (5.00)	27 (3.08)
Marital status	Single	18 (1.92)	6 (10.00)	12 (1.37)
	Married	826 (88.25)	48 (80.00)	778 (88.61)
	Divorced	3 (0.32)	1 (1.67)	2 (0.23)
	In a relationship (non-married)	85 (9.08)	4 (6.67)	81 (9.23)
	Widowed	1 (0.11)	1 (1.67)	0 (0)
	Other	3 (0.32)	0 (0)	3 (0.34)
Sexual orientation	Heterosexual or straight	793 (84.54)	56 (93.33)	737 (83.94)
	Bisexual	101 (10.77)	3 (5.00)	98 (11.16)
	Homosexual	18 (1.92)	0	18 (2.05)
	Pansexual	19 (2.03)	0	19 (2.16)
	Asexual	7 (0.75)	1 (1.67)	6 (0.68)
Level of education	High school degree or equivalent	73 (7.78)	8 (13.33)	65 (7.40)
	Bachelor’s degree	390 (41.58)	29 (48.33)	361 (41.12)
	Master’s degree	295 (31.45)	10 (16.67)	285 (32.46)
	Doctorate degree	114 (12.15)	6 (10.00)	8 (0.91)
	Other	66 (7.04)	7 (11.67)	59 (6.72)
Household income	< $24,999	11 (1.18)	3 (5.00)	8 (0.91)
	$25,000-$49,999	57 (6.09)	3 (5.00)	54 (6.16)
	$50,000-$74,999	108 (11.54)	9 (15.00)	99 (11.30)
	$75,000-$99,999	145 (15.49)	6 (10.00)	139 (15.87)
	$100,000-$149,999	258 (27.56)	22 (36.67)	236 (26.94)
	> $150,000	357 (38.14)	17 (28.33)	340 (38.81)

1,018 individuals participated in this study with 938 adequately completing the survey. A total of 878 females and 60 males participated in our study. The majority of respondents were White, between ages 30-35, married, heterosexual, with a bachelor’s degree as the highest level of education and household income above $100,000 amongst both females and females.

**Figure 1:**
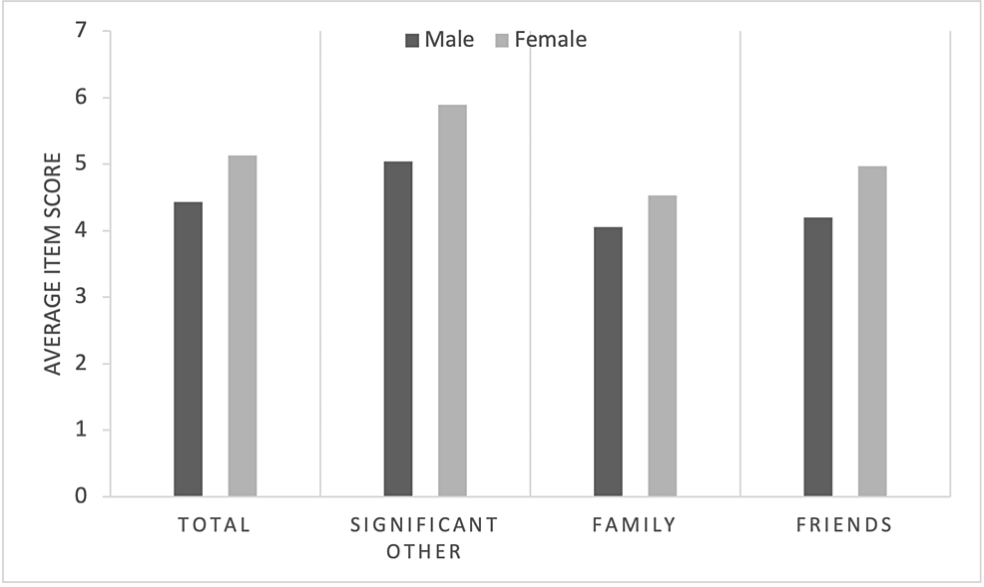
Clustered Bar Chart of Mean Scores by Subscale

Data are mean ± standard deviation, unless otherwise stated. Mean total scores were higher in women (5.13 ± 1.10) compared with men (4.43 ± 1.58). Mean scores on all subscales (SO, family, friend) were higher in women (5.90 ± 1.20, 4.53 ± 1.53, 4.97 ± 1.40) compared with men (5.04 ± 1.90, 4.06 ± 1.76, 4.20 ± 1.81), respectively (Table 3, Figure 1).

**Table 3:** Group Descriptive Statistics

	Factor	Mean	Standard Deviation (SD)	Minimum	Maximum
Male	Total	4.43	1.58	1	6.666667
	Significant Other	5.04	1.90	1	7
	Family	4.06	1.76	1	6.75
	Friends	4.20	1.81	1	7
Female	Total	5.13	1.11	1	7
	Significant Other	5.90	1.20	1	7
	Family	4.53	1.53	1	7
	Friends	4.97	1.40	1	7

**Figure 2:**
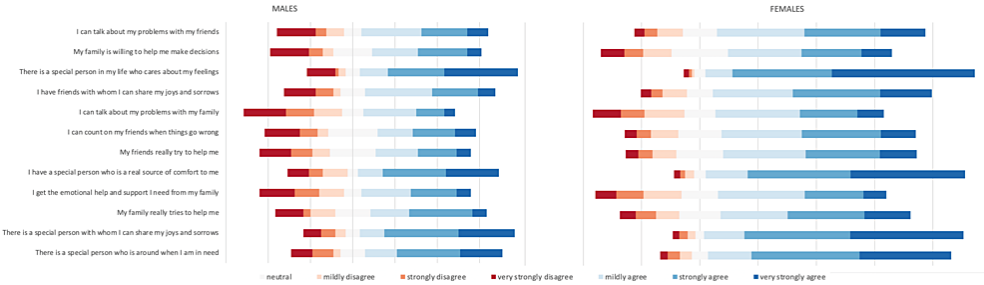
Multidimensional Scale of Perceived Social Support Responses by Gender

A Welch t-test was run to determine if there were differences in scores on the MSPSS subscales (total, SO, family, friend) between males and females, due to the assumption of homogeneity of variances being violated, as assessed by Levene’s test for equality of variances (p < 0.001, p < 0.001, p= 0.013, p < 0.001, respectively). There were outliers in the data, as assessed by inspection of a boxplot, however, it was determined that the outliers would have no significant effect as results were similar when testing both with and without the inclusion of outliers. Scores for all subscales were approximately normally distributed, as assessed by visual inspection of a Normal Q-Q Plot, which is the most preferable method in the setting of large sample sizes. There was a statistically significant difference in mean total score between females and males, with females scoring higher than males, 0.70 (95% CI, -1.11 to -0.28), t(63.018) = -3.360, p = .001. There was a statistically significant difference in mean SO score between females and males, with females scoring higher than males, 0.86 (95% CI, -1.35 to -0.36), t(62.277) = -3.452, p = .001. There was a statistically significant difference in mean family score between females and males, with females scoring higher than males, 0.47 (95% CI, -0.94 to -0.01), t(65.219) = -2.039, p = 0.046. There was a statistically significant difference in mean friend score between females and males, with females scoring higher than males, 0.76 (95% CI, -1.24 to -0.29), t(63.018) = -3.360, p = 0.002 (Table 4). Figure 2 provides a visual representation of the breakdown of individual item scores within the MSPSS for males and females. 

**Table 4:** Independent Samples Test SO: significant other

		Levene's Test for Equality of Variances									
		F	Sig.	t	df	Significance		Mean Difference	Std. Error Difference	95% Confidence Interval of the Difference	
						One-Sided p	Two-Sided p			Lower	Upper
Total score	Equal variances assumed	29.622	< .001	-4.573	935	< .001>	< .001>	-.69812	.15266	-.99772	-.39851
	Equal variances not assumed			-3.360	63.018	< .001>	.001	-.69812	.20777	-1.11332	-.28292
SO score	Equal variances assumed	38.259	< .001	-5.111	935	< .001>	< .001>	-.85628	.16753	-1.18505	-.52751
	Equal variances not assumed			-3.452	62.277	< .001>	.001	-.85628	.24806	-1.35211	-.36045
Family score	Equal variances assumed	6.242	.013	-2.308	935	.011	.021	-.47473	.20571	-.87844	-.07103
	Equal variances not assumed			-2.039	65.219	.023	.046	-.47473	.23285	-.93975	-.00972
Friend score	Equal variances assumed	11.565	< .001	-3.991	935	< .001>	< .001>	-.76334	.19125	-1.13867	-.38800
	Equal variances not assumed			-3.205	63.966	.001	.002	-.76334	.23815	-1.23910	-.28757

As our sample sizes were largely unequal, we additionally repeated our analysis with a random sampling of the larger group (females) to create a more similarly sized comparison group for the smaller group (males), which resulted in similar results. Specifically, we compared 60 male and 64 female participants. Mean total scores were higher in women (5.21 ± 1.09) compared with men (4.43 ± 1.58). Mean scores on all subscales (SO, family, friend) were higher in women (5.88 ± 1.19, 4.66 ± 1.47, 5.10 ± 1.34) compared with men (5.04 ± 1.90, 4.06 ± 1.76, 4.20 ± 1.81), respectively (Table 5). Our findings regarding outliers and normality were the same as described above. As the assumption of homogeneity of variances was again violated, as assessed by Levene’s test for equality of variances (p <0.001, p =0.020, p = 0.005, p < 0.001, respectively), a Welch t-test was run. There was a statistically significant difference in mean total score between females and males, with females scoring higher than males, 0.78 (95% CI, -1.26 to -0.30), t(103.932) = -3.170, p = .002. There was a statistically significant difference in mean SO score between females and males, with females scoring higher than males, 0.84 (95% CI, -1.40 to -0.27), t(97.859) = -2.93, p = .004. There was a statistically significant difference in mean family score between females and males, with females scoring higher than males, 0.60 (95% CI, -1.18 to -0.02), t(115.350) = -2.059, p = 0.042. There was a statistically significant difference in mean friend score between females and males, with females scoring higher than males, 0.90 (95% CI, -1.47 to -0.33), t(108.506) = -3.123, p = 0.002 (Table 6).

**Table 5:** Group Descriptive Statistics SO: significant other

	sex	N	Mean	Std. Deviation
Total score	male	60	4.4347	1.58308
	female	64	5.2135	1.09087
Family score	male	60	4.0583	1.75898
	female	64	4.6602	1.47221
Friend score	male	60	4.2042	1.80776
	female	64	5.1016	1.34055
SO score	male	60	5.0417	1.89567
	female	64	5.8789	1.18522

**Table 6:** Independent Samples Test SO: Significant Other

		Levene's Test for Equality of Variances							
		F	Sig.	t	df	95% Confidence Interval of the Difference			
						One-Sided p	Lower	Upper	
Total score	Equal variances assumed	14.456	< .001>	-3.207	122	< .001>	-1.25958	-.29805	
	Equal variances not assumed			-3.170	103.932	.001	-1.26603	-.29161	
Family score	Equal variances assumed	5.590	.020	-2.071	122	.020	-1.17713	-.02651	
	Equal variances not assumed			-2.059	115.350	.021	-1.18077	-.02288	
Friend score	Equal variances assumed	8.274	.005	-3.153	122	.001	-1.46080	-.33399	
	Equal variances not assumed			-3.123	108.506	.001	-1.46686	-.32793	
SO score	Equal variances assumed	15.817	< .001>	-2.969	122	.002	-1.39555	-.27892	
	Equal variances not assumed			-2.927	97.859	.002	-1.40497	-.26951	

A two-way ANOVA was conducted on a random smaller sub-sample of individuals who provided their infertility diagnoses to examine the effects of gender and infertility diagnosis on total score. Data are mean ± standard deviation, unless otherwise stated. Residual analyses for each subscale were performed to test for the assumptions of the two-way ANOVA. Outliers were assessed by inspection of a boxplot, normality was assessed using Shapiro-Wilk's normality test for each cell of the design and homogeneity of variances was assessed by Levene's test. There was one outlier in the data, however, it was determined that the outlier would have no significant effect as results were similar when testing both with and without the inclusion of the outlier. Residuals were normally distributed (p > .05) and there was homogeneity of variances (p = 0.085).

There was a statistically significant interaction between sex and infertility diagnosis for "Total score,” F(1, 22) = 6.404, p = .019, partial η2 = 0.225. Therefore, an analysis of simple main effects for gender and for infertility diagnosis was performed with statistical significance receiving a Bonferroni adjustment and being accepted at the p < .025 level.

There was a statistically significant difference in mean “total score” for males with either male-factor or female-factor infertility, F(1,22) = 9.638, p < .005, partial η2 = .305, but not for females, F(1, 22) = 0.225, p = 0.640, partial η2 = 0.010 (Table 7). All pairwise comparisons were run for each simple main effect with reported 95% confidence intervals and p-values Bonferroni-adjusted within each simple main effect. Mean “total scores” for males with male-factor and female-factor infertility were 2.93 ± 1.62 and 5.15 ± 1.28, respectively. Males with male-factor infertility had a statistically significantly lower mean total score than males with female-factor infertility, -2.22 (95% CI, -3.71 to -0.74), p= 0.005. Mean total scores for females with male-factor and female-factor infertility were 5.66 ± 0.80 and 5.32 ± 1.15, respectively. There was no statistically significant difference in mean total scores among females with male-factor and female-factor infertility, 0.34 (95% CI, -1.15 to 1.82), p= 0.640 (Table 8). 

**Table 7:** Univariate Tests for Total Score

Sex		Sum of Squares	df	Mean Square	F	Sig.	Partial Eta Squared
male	Contrast	15.204	1	15.204	9.638	.005	.305
	Error	34.704	22	1.577			
female	Contrast	.355	1	.355	.225	.640	.010
	Error	34.704	22	1.577			

**Table 8:** Pairwise Comparisons for Total Score MFI= male-factor infertility, FFI= female-factor infertility *The mean difference is significant at the .05 level; ^b^Adjustment for multiple comparisons: Bonferroni

sex	(I) infertility	(J) infertility	Mean Difference (I-J)	Std. Error	Sig.^b^	95% Confidence Interval for Difference^b^	
						Lower Bound	Upper Bound
male	MFI	FFI	-2.223^*^	.716	.005	-3.708	-.738
	FFI	MFI	2.223^*^	.716	.005	.738	3.708
female	MFI	FFI	.340	.716	.640	-1.145	1.824
	FFI	MFI	-.340	.716	.640	-1.824	1.145

For males and females with male-factor infertility, mean “total” score was 2.73 (95% CI, 1.43 to 4.03) points lower for males than females, F(1,22) = 18.89, p < 0.001, partial η2 = 0.462. In contrast, for males and females with female-factor infertility, mean “total” score was 0.167 (95% CI, -1.81 to 1.48) points lower in males than females, F (1, 22) = 0.044, p < 0.836, partial η2 = 0.002, which was not significantly different (Table 9, Table 10).

**Table 9:** Univariate Tests for Total Score MFI= male-factor infertility, FFI= female-factor infertility

infertility		Sum of Squares	df	Mean Square	F	Sig.	Partial Eta Squared
MFI	Contrast	29.793	1	29.793	18.887	< .001	.462
	Error	34.704	22	1.577			
FFI	Contrast	.069	1	.069	.044	.836	.002
	Error	34.704	22	1.577			

**Table 10:** Pairwise Comparisons for Total Score MFI= male-factor infertility, FFI= female-factor infertility *The mean difference is significant at the .05 level; ^b^Adjustment for multiple comparisons: Bonferroni

infertility	(I) sex	(J) sex	Mean Difference (I-J)	Std. Error	Sig.^b^	95% Confidence Interval for Difference^b^	
						Lower Bound	Upper Bound
MFI	male	female	-2.729^*^	.628	< .001>	-4.032	-1.427
	female	male	2.729^*^	.628	< .001>	1.427	4.032
FFI	male	female	-.167	.794	.836	-1.814	1.481
	female	male	.167	.794	.836	-1.481	1.814

## Discussion

Our findings contribute to a growing body of literature that examines gender differences in perceived social support in couples experiencing issues with fertility. In a comparison of MSPSS scores between males and females, we found higher mean scores across all subscales in females. Numerical scores on each subscale correspond with the following categories: low social support is defined as a score of 1-2.9, moderate as 3-5 and high as 5.1-7. Females were found to have high levels of total and SO perceived support, and moderate levels of family and friend support. In comparison, males had high levels of SO support and moderate levels of total, family, and friend support. The biggest difference between sexes was seen with SO and friend support, with the smallest difference being seen with family support.

While these findings are inconsistent with a majority of existing literature that found no statistically significant gender differences in perceived social support [18,20], several studies have found higher levels of social support in women than in men [18,21-23].

Hosseini et al argue that these differences in social support indicate that women may be considerably more affected by the issue of infertility than men [23], however, we suggest an alternative explanation. Previous studies have found that lower overall scores in males may be associated with lower levels of social support seeking and disclosure to others [17,24-25]. These findings may be related to the profoundly adverse impact that infertility has on masculinity and the more markedly stigmatizing nature of male infertility as men may often conflate infertility, virility and sexual potency which can therefore lead to perceived personal inadequacy [17,26].

Research on North American couples has shown that men struggling with infertility tend to use fewer coping strategies overall and are less apt to use social support [6] or explicitly seek it out and spontaneously disclose their emotional needs [27]. Findings from a Swedish study found that a large proportion of males had not confided in anyone other than their spouse, which is consistent with our findings suggestive of SO as the highest source of social support in males [25,27]. Males diagnosed as infertile were found to avoid disclosure with anyone other than their wives largely due to shame about the diagnosis [27,28]. While it was found that men experience intense ‘emotional anguish’ as a result of infertility, they reported a need for this to be suppressed so as to protect their partners. In similar regard, online support groups, which do not involve face-to-face encounters, were suggested as a possibly useful tool for men to confide in others [27,29]. These findings merely demonstrate a different means of coping in men, but do not necessarily mean that men don’t need the support [26] as men appear to be at least equally affected, if not more affected, by infertility, and perhaps even neglected. It may thus be advantageous for clinicians to explicitly inquire and assess male mental health to detect psychological symptoms. A statement and question such as, “I have met many men experiencing infertility, and they often feel sad, worried, embarrassed, lonely, etc. ... have you had any feelings of this kind [27]?” may be a useful approach to do so.

When comparing scores with respect to infertility diagnoses, males with male-factor infertility showed significantly lower total scores compared to males with female-factor infertility. This finding is consistent with those of Cousinaeu and Domar [6] and Nachtigall et al [30] and is likely related to the aforementioned negative emotional responses and increased stigma associated with male-factor infertility, as is related to perceived masculinity. In contrast, total scores with respect to infertility diagnoses did not differ in females. This may suggest generalized distress over infertility in women, regardless of cause, in contrast to men, where concerns may be amplified or most visible when directly related to the male factor. Alternatively, these findings may simply highlight heightened distress when it is due to the male factor, relative to the already high levels of distress brought on by infertility.

Additionally, amongst males and females with a male-factor infertility diagnosis, total scores were significantly lower in males compared with females, whereas amongst males and females with a female-factor infertility diagnosis, total scores were not statistically significantly different.

Research concerning the psychosocial aspects of infertility and treatment more often focuses on women than men [27]. Our findings suggest that men experience significant psychological repercussions of infertility, highlighting the importance of focusing on the male experience that seems to be under-represented in the literature [6].

A significant strength of our study is seen in our sampling methods as we sampled an online support group rather than a clinic-based population. A majority of previous research relies too heavily on convenience samples drawn from a patient population which is certainly not representative, as this leaves out a large portion of the infertile population - those that don’t seek treatment. That same over-reliance on clinic samples forces a focus on infertility patients rather than on infertility people which makes it difficult to separate the psychological consequences of infertility from the psychological consequences of treatment [22].

Additionally, our male and female samples were not drawn from the infertile couple unit but rather independently. It has been shown that couples' scores on distress scales are correlated and therefore studies which use couple samples for gender comparison may be under-reporting the real extent of the differences between genders [22]. Examining these differences among couples, or the “shared experience” of infertility, may lead to a narrowing of the gender gap. That is, men and women that are together may be more similar than different in their perceptions of social support [17].

Our study had several limitations. Firstly, individuals may have had varying interpretations of what was meant by infertility or by their specific diagnoses. As infertility is an issue that affects both individuals, individuals may have chosen “both male and female-factor” even if the true diagnosis was exclusively “male” or “female-factor.” Further, as a distressing and emotional issue, individuals may consider themselves infertile based on their own definition, even when formally, they don’t meet the criteria required for that diagnosis. While these issues could be adjusted for those individuals that provided their experiences or commentaries at the end of the survey, not all individuals decided to do so, thereby leaving many data points ambiguous. Additionally, we had a limited male sample size. Although this is not surprising given males’ general hesitancy for disclosure, those that did respond may have had different characteristics and/ or sentiments compared with non-responders. While this may make it more difficult to generalize/limits generalizability, our preliminary findings introduce important and overlooked findings regarding the male experience with infertility. Future studies would thus benefit from a larger sample size of males to both further investigate our findings and also to gain more insights and perspectives on the infertile male experience.

## Conclusions

In conclusion, we found significantly lower levels of perceived social support in males experiencing issues with infertility as compared with females. Moreover, males with male-factor infertility had lower scores than females with the same diagnosis, or females with female-factor infertility. Given the implications of high levels of perceived social support on improved overall well-being, our findings highlight the importance of implementing interventions that are focused on improving perceptions of social support in males experiencing issues with infertility, with specific consideration given to the unique experiences/challenges and factors that may impact their experience.

## References

[1] Ombelet W (2020). WHO fact sheet on infertility gives hope to millions of infertile couples worldwide. Facts Views Vis Obgyn.

[2] Hocaoglu C The psychosocial aspect of infertility. Infertility, Assisted Reproductive Technologies and Hormone Assays.

[3] Patel A, Sharma PS, Narayan P, Binu VS, Dinesh N, Pai PJ (2016). Prevalence and predictors of infertility-specific stress in women diagnosed with primary infertility: a clinic-based study. J Hum Reprod Sci.

[4] Ezzell W (2016). The impact of infertility on women's mental health. N C Med J.

[5] Rooney KL, Domar AD (2018). The relationship between stress and infertility. Dialogues Clin Neurosci.

[6] Cousineau TM, Domar AD (2007). Psychological impact of infertility. Best Pract Res Clin Obstet Gynaecol.

[7] Jirka J, Schuett S, Foxall MJ (1996). Loneliness and social support in infertile couples. J Obstet Gynecol Neonatal Nurs.

[8] Saleem S, Qureshi NS, Mahmood Z (2019). Attachment, perceived social support and mental health problems in women with primary infertility. Int J Reprod Contracep Obstetr Gynecol.

[9] Taylor SE, Seeman TE (1999). Psychosocial resources and the SES-health relationship. Ann N Y Acad Sci.

[10] Slade P, O'Neill C, Simpson AJ, Lashen H (2007). The relationship between perceived stigma, disclosure patterns, support and distress in new attendees at an infertility clinic. Hum Reprod.

[11] Martins MV, Peterson BD, Almeida V, Mesquita-Guimarães J, Costa ME (2014). Dyadic dynamics of perceived social support in couples facing infertility. Hum Reprod.

[12] Eagle DE, Hybels CF, Proeschold-Bell RJ (2018). Perceived social support, received social support, and depression among clergy. J Soc Personal Relation.

[13] Coventry WL, Gillespie NA, Heath AC, Martin NG (2004). Perceived social support in a large community sample--age and sex differences. Soc Psychiatry Psychiatr Epidemiol.

[14] Wang J, Mann F, Lloyd-Evans B, Ma R, Johnson S (2018). Associations between loneliness and perceived social support and outcomes of mental health problems: a systematic review. BMC Psychiatry.

[15] Petok WD (2006). The psychology of gender-specific infertility diagnoses. Infertility Counseling: A Comprehensive Handbook for Clinicians.

[16] Mikkelsen AT, Madsen SA, Humaidan P (2013). Psychological aspects of male fertility treatment. J Adv Nurs.

[17] Jordan C, Revenson TA (1999). Gender differences in coping with infertility: a meta-analysis. J Behav Med.

[18] Ying LY, Wu LH, Loke AY (2015). Gender differences in experiences with and adjustments to infertility: a literature review. Int J Nurs Stud.

[19] Zimet GD, Dahlem NW, Zimet SG, Farley GK (1988). The multidimensional scale of perceived social support. J Personal Assess.

[20] Karlidere T, Bozkurt A, Yetkin S, Doruk A, Sütçigil L, Nahit Ozmenler K, Ozşahin A (2007). [Is there gender difference in infertile couples with no axis one psychiatric disorder in context of emotional symptoms, social support and sexual function?]. Turk Psikiyatri Derg.

[21] Abbey A, Halman LJ, Andrews FM (1992). Psychosocial, treatment, and demographic predictors of the stress associated with infertility. Fertil Steril.

[22] Greil AL, Slauson-Blevins K, McQuillan J (2010). The experience of infertility: a review of recent literature. Sociol Health Illn.

[23] Hosseini M, Sepidarkish M, Omani-Samani R, Maroufizadeh S (2021). Gender differences in self-efficacy, resilience, and social support among infertile iranian couples: a dyadic approach. Iran J Public Health.

[24] Soman S, Bhat SM, Latha KS, Praharaj SK (2016). Gender differences in perceived social support and stressful life events in depressed patients. East Asian Arch Psychiatry.

[25] Hjelmstedt A, Andersson L, Skoog-Svanberg A, Bergh T, Boivin J, Collins A (1999). Gender differences in psychological reactions to infertility among couples seeking IVF- and ICSI-treatment. Acta Obstet Gynecol Scand.

[26] Dudgeon MR, Inhorn MC (2003). Gender, masculinity, and reproduction: anthropological perspectives. Int J Men's Health.

[27] Fisher JR, Hammarberg K (2012). Psychological and social aspects of infertility in men: an overview of the evidence and implications for psychologically informed clinical care and future research. Asian J Androl.

[28] Lee TY, Chu TY (2001). The Chinese experience of male infertility. West J Nurs Res.

[29] Malik S, Coulson N (2008). The male experience of infertility: a thematic analysis of an online infertility support group bulletin board. J Reprod Infant Psychol.

[30] Nachtigall RD, Becker G, Wozny M (1992). The effects of gender-specific diagnosis on men's and women's response to infertility. Fertil Steril.

