# Correction: Possible Gender Differences in the Level of Perceived Social Support in Couples Who Are Experiencing Issues With Infertility

**DOI:** 10.7759/cureus.c127

**Published:** 2023-07-07

**Authors:** Maya Pinzon, Shawna Rotoli

**Affiliations:** 1 Department of Molecular Biology, Rowan University School of Osteopathic Medicine, Stratford, USA

This article has been corrected at the request of the submitting author to change the name Maya Borowczak to Maya Pinzon.

